# Diaqua­bis­(dihydrogen 3-aza­niumyl-1-hy­droxy­propyl­idene-1,1-di­phos­phon­ato-κ^2^ *O*,*O*′)cobalt(II)

**DOI:** 10.1107/S1600536811045120

**Published:** 2011-11-05

**Authors:** Natalia V. Tsaryk, Anatolij V. Dudko, Alexandra N. Kozachkova, Vasily I. Pekhnyo

**Affiliations:** a Institute of General and Inorganic Chemistry, NAS Ukraine, Kyiv, prosp. Palladina 32/34, 03680, Ukraine

## Abstract

The asymmetric unit of title compound, [Co(C_3_H_10_NO_7_P_2_)_2_(H_2_O)_2_], contains one half-mol­ecule of the complex. The Co^II^ atom is located on an inversion centre and displays a distorted octa­hedral coordination geometry defined by four O atoms of two 3-aza­niumyl-1-hy­droxy­propyl­idene-1,1-bis­phospho­nato ligands in the equatorial plane and two water mol­ecules located in axial positions. The ligand mol­ecules, which exist in a zwitterionic state, form two six-membered chelate rings with chair conformations. In the crystal, mol­ecules are inter­linked by O—H⋯O and N—H⋯O hydrogen bonds, forming a three-dimensional supra­molecular structure.

## Related literature

For general background to organic diphospho­nic acids and their applications, see: Matczak-Jon & Videnova-Adrabinska (2005 ▶). For applications of bis­phospho­nate metal complexes in medicine, see: Matkovskaya *et al.* (2001 ▶). For a related structure, see: Bon *et al.* (2010 ▶). For bond-length data, see: Allen *et al.* (2004 ▶).
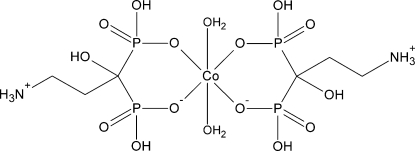


## Experimental

### 

#### Crystal data

[Co(C_3_H_10_NO_7_P_2_)_2_(H_2_O)_2_]
*M* *_r_* = 563.08
Monoclinic, 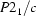

*a* = 7.3292 (2) Å
*b* = 10.8172 (3) Å
*c* = 12.6403 (3) Å
β = 120.801 (1)°
*V* = 860.79 (4) Å^3^
*Z* = 2
Mo *K*α radiation
μ = 1.46 mm^−1^
*T* = 100 K
0.50 × 0.25 × 0.15 mm

#### Data collection

Bruker APEXII CCD diffractometer
Absorption correction: multi-scan (*SADABS*; Bruker, 2007 ▶) *T*_min_ = 0.528, *T*_max_ = 0.811
6688 measured reflections
2613 independent reflections
2273 reflections with *I* > 2σ(*I*)
*R*_int_ = 0.020

#### Refinement

*R*[*F* ^2^ > 2σ(*F* ^2^)] = 0.029
*wR*(*F* ^2^) = 0.080
*S* = 1.04
2613 reflections
157 parameters
1 restraint
H atoms treated by a mixture of independent and constrained refinement
Δρ_max_ = 0.70 e Å^−3^
Δρ_min_ = −0.41 e Å^−3^

### 

Data collection: *APEX2* (Bruker, 2007 ▶); cell refinement: *SAINT* (Bruker, 2007 ▶); data reduction: *SAINT*; program(s) used to solve structure: *SHELXS97* (Sheldrick, 2008 ▶); program(s) used to refine structure: *SHELXL97* (Sheldrick, 2008 ▶); molecular graphics: *DIAMOND* (Brandenburg & Putz, 2010 ▶); software used to prepare material for publication: *publCIF* (Westrip, 2010 ▶).

## Supplementary Material

Crystal structure: contains datablock(s) I, global. DOI: 10.1107/S1600536811045120/ez2263sup1.cif

Structure factors: contains datablock(s) I. DOI: 10.1107/S1600536811045120/ez2263Isup2.hkl

Additional supplementary materials:  crystallographic information; 3D view; checkCIF report

## Figures and Tables

**Table 1:** Hydrogen-bond geometry (Å, °)

*D*—H⋯*A*	*D*—H	H⋯*A*	*D*⋯*A*	*D*—H⋯*A*
O1—H1*O*⋯O5^i^	0.85 (3)	2.05 (3)	2.845 (2)	155 (2)
O3—H3*O*⋯O7^i^	0.76 (3)	1.72 (3)	2.4627 (19)	167 (3)
O6—H6*O*⋯O4^ii^	0.74 (3)	1.78 (3)	2.5124 (18)	175 (3)
O8—H81⋯O4^iii^	0.89 (3)	1.88 (3)	2.7277 (18)	160 (2)
O8—H82⋯O1^iv^	0.77 (3)	2.25 (3)	2.8953 (19)	142 (3)
N1—H2*N*⋯O6^iv^	0.88 (3)	2.15 (3)	2.975 (2)	156 (2)
N1—H3*N*⋯O2^iii^	0.91 (3)	2.30 (3)	3.096 (2)	146 (2)
N1—H3*N*⋯O5^v^	0.91 (3)	2.32 (3)	3.030 (2)	134 (2)
N1—H1*N*⋯O4^vi^	0.87 (2)	2.33 (2)	3.071 (2)	143 (2)

Symmetry codes: (i) 
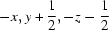
; (ii) 
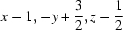
; (iii) 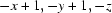; (iv) 
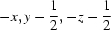
; (v) 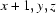; (vi) 
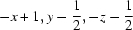
.

**Fig. 1.**
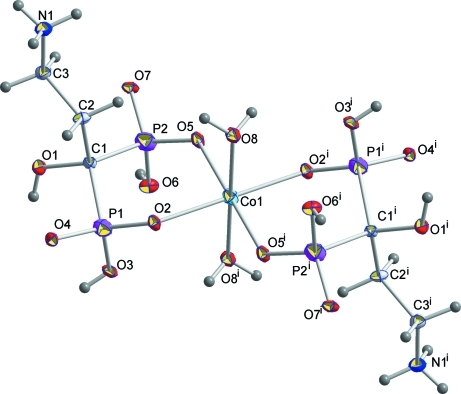
The molecular structure of the title compound. Displacement ellipsoids are drawn at the 50% probability level.

**Fig. 2.**
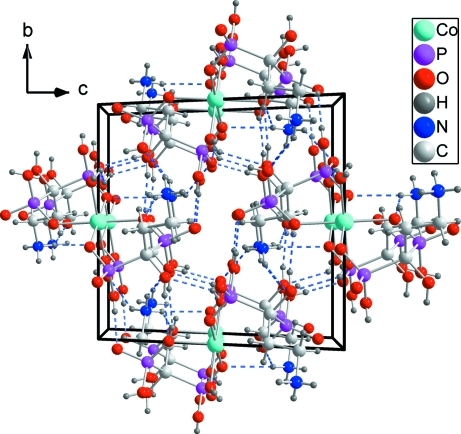
Crystal packing of the title compound viewed down the a axis, showing the three-dimensional chain structure. Hydrogen bonds are shown as dashed lines.

## supplementary crystallographic information

### Comment

In recent years the design and synthesis of novel metal-organic coordination compounds based on gem-diphosphonic acids has attracted much interest due to their structural diversity and possible applications in many areas (Matczak-Jon & Videnova-Adrabinska, 2005). Particular attention has been paid to 3-amino-1-hydroxypropane-1,1-diyl)bis(phosphonic acid) (pamidronic acid) due to its biological activity and as a result of its usage as a drug to prevent calcification and inhibit bone resorption, *etc*. (Matkovskaya *et al.*, 2001).

The molecular structure of title complex is shown in Fig. 1; as illustrated the molecule of the complex forms discrete monomeric units. The asymmetric unit contains one-half of the formula unit [Co(C_3_H_10_NO_7_P_2_)_2_(H_2_O)_2_], with the Co atom lying on an inversion center.

The Co^II^ ion is coordinated in a slightly distorted octahedral geometry which consists of six oxygen atoms, two from water molecules located in the axial positions and four from the two phosphonate groups of two different ligands, which exist in zwitterionic form, creating two six-membered [O, O] chelate rings. The Co—O bond lengths and the O—Co—O angles have expected values (Allen *et al.*, 2004) and conform well to the previously reported related structure (Bon *et al.*, 2010). In the packing, O—H···O and N—H···O hydrogen-bonds exist between the water molecules, phosphonate, hydroxyl O atoms and nitrogen atoms of the amino group (Fig. 2, Table 1). Thus, the molecules are interlinked by these hydrogen bonds to create a three-dimensional structure which partially influences and stabilizes the configuration of the molecule.

### Experimental

Light pink crystals of the title compound were obtained from a mixture of Co(NO_3_)_2_.6H_2_O (0,25 mmol; 0,0728 g) and 3-aminohydroxypropilidene-1,1-diphosphonic acid (0,5 mmol; 0,1175 g) in 20 ml H_2_O. The combined solution was allowed to slowly evaporate. After 30 days, suitable crystals for X-ray data collection were obtained.

### Refinement

All non-H atoms were refined with anisotropic displacement parameters. H atoms bonded to O and N atoms were located in a difference Fourier map. Their positions were refined freely whereas displacement parameters were fixed to *U*_iso_(H) = 1.5*U*_eq_(N,*O*).The H1n atom of the amino group was refined with a distance restraint (N—H= 0.91 Å). Other H atoms bonded to C were positioned geometrically and refined using a riding model with C—H = 0.99 Å for CH_2_ with *U*_iso_(H) = 1.2*U*_eq_(C).

### Crystal data

**Table:**

[Co(C_3_H_10_NO_7_P_2_)_2_(H_2_O)_2_]	*F*(000) = 578
*M**_r_* = 563.08	*D*_x_ = 2.172 Mg m^−^^3^
Monoclinic, *P*2_1_/*c*	Mo *K*α radiation, λ = 0.71073 Å
Hall symbol: -P 2ybc	Cell parameters from 3543 reflections
*a* = 7.3292 (2) Å	θ = 2.7–30.6°
*b* = 10.8172 (3) Å	µ = 1.46 mm^−^^1^
*c* = 12.6403 (3) Å	*T* = 100 K
β = 120.801 (1)°	Block, pink
*V* = 860.79 (4) Å^3^	0.50 × 0.25 × 0.15 mm
*Z* = 2	

### Data collection

**Table:**

Bruker APEXII CCD diffractometer	2613 independent reflections
Radiation source: fine-focus sealed tube	2273 reflections with *I* > 2σ(*I*)
graphite	*R*_int_ = 0.020
φ and ω scans	θ_max_ = 30.6°, θ_min_ = 2.7°
Absorption correction: multi-scan (*SADABS*; Bruker, 2007)	*h* = −10→9
*T*_min_ = 0.528, *T*_max_ = 0.811	*k* = −12→15
6688 measured reflections	*l* = −18→12

### Refinement

**Table:**

Refinement on *F*^2^	Primary atom site location: structure-invariant direct methods
Least-squares matrix: full	Secondary atom site location: difference Fourier map
*R*[*F*^2^ > 2σ(*F*^2^)] = 0.029	Hydrogen site location: inferred from neighbouring sites
*wR*(*F*^2^) = 0.080	H atoms treated by a mixture of independent and constrained refinement
*S* = 1.04	*w* = 1/[σ^2^(*F*_o_^2^) + (0.0452*P*)^2^ + 0.4224*P*] where *P* = (*F*_o_^2^ + 2*F*_c_^2^)/3
2613 reflections	(Δ/σ)_max_ = 0.003
157 parameters	Δρ_max_ = 0.70 e Å^−^^3^
1 restraint	Δρ_min_ = −0.41 e Å^−^^3^

### Special details

**Table:**

Geometry. All e.s.d.'s (except the e.s.d. in the dihedral angle between two l.s. planes) are estimated using the full covariance matrix. The cell e.s.d.'s are taken into account individually in the estimation of e.s.d.'s in distances, angles and torsion angles; correlations between e.s.d.'s in cell parameters are only used when they are defined by crystal symmetry. An approximate (isotropic) treatment of cell e.s.d.'s is used for estimating e.s.d.'s involving l.s. planes.
Refinement. Refinement of *F*^2^ against ALL reflections. The weighted *R*-factor *wR* and goodness of fit *S* are based on *F*^2^, conventional *R*-factors *R* are based on *F*, with *F* set to zero for negative *F*^2^. The threshold expression of *F*^2^ > σ(*F*^2^) is used only for calculating *R*-factors(gt) *etc*. and is not relevant to the choice of reflections for refinement. *R*-factors based on *F*^2^ are statistically about twice as large as those based on *F*, and *R*- factors based on ALL data will be even larger.

### Fractional atomic coordinates and isotropic or equivalent isotropic displacement parameters (Å^2^)

**Table:**

	*x*	*y*	*z*	*U*_iso_*/*U*_eq_	
Co1	0.0000	0.5000	0.0000	0.01045 (9)	
P1	0.25936 (6)	0.70145 (4)	−0.05843 (4)	0.00902 (10)	
P2	−0.12574 (6)	0.58120 (4)	−0.28208 (4)	0.00964 (10)	
O1	0.1630 (2)	0.72770 (13)	−0.29229 (12)	0.0156 (3)	
H1O	0.127 (4)	0.802 (3)	−0.289 (2)	0.023*	
O2	0.25165 (18)	0.60515 (12)	0.02492 (11)	0.0124 (2)	
O3	0.1139 (2)	0.81197 (12)	−0.07370 (12)	0.0145 (3)	
H3O	0.154 (4)	0.874 (3)	−0.079 (2)	0.022*	
O4	0.48210 (18)	0.74524 (13)	−0.01964 (11)	0.0137 (3)	
O5	−0.1323 (2)	0.49001 (12)	−0.19349 (11)	0.0143 (3)	
O6	−0.2632 (2)	0.69798 (12)	−0.29892 (12)	0.0151 (3)	
H6O	−0.334 (4)	0.712 (2)	−0.365 (2)	0.023*	
O7	−0.1968 (2)	0.52889 (12)	−0.40857 (11)	0.0137 (3)	
O8	0.1663 (2)	0.33228 (13)	0.02254 (13)	0.0158 (3)	
H81	0.270 (4)	0.320 (2)	0.007 (2)	0.024*	
H82	0.082 (4)	0.283 (2)	−0.014 (2)	0.024*	
N1	0.4212 (3)	0.38826 (17)	−0.30480 (17)	0.0192 (3)	
H2N	0.356 (4)	0.323 (3)	−0.297 (2)	0.029*	
H3N	0.553 (5)	0.394 (2)	−0.237 (3)	0.029*	
H1N	0.439 (4)	0.383 (3)	−0.3675 (19)	0.029*	
C1	0.1504 (2)	0.63551 (16)	−0.21320 (15)	0.0108 (3)	
C2	0.2914 (3)	0.52507 (17)	−0.20331 (16)	0.0140 (3)	
H2A	0.4370	0.5391	−0.1330	0.017*	
H2B	0.2357	0.4493	−0.1858	0.017*	
C3	0.3018 (3)	0.50443 (17)	−0.31959 (18)	0.0164 (4)	
H3A	0.1565	0.4980	−0.3922	0.020*	
H3B	0.3743	0.5750	−0.3325	0.020*	

### Atomic displacement parameters (Å^2^)

**Table:**

	*U*^11^	*U*^22^	*U*^33^	*U*^12^	*U*^13^	*U*^23^
Co1	0.01100 (15)	0.00938 (17)	0.00988 (16)	−0.00047 (11)	0.00457 (12)	0.00097 (12)
P1	0.00920 (18)	0.0085 (2)	0.00885 (19)	−0.00167 (14)	0.00423 (15)	−0.00129 (15)
P2	0.01040 (19)	0.0090 (2)	0.00774 (18)	−0.00077 (14)	0.00333 (15)	−0.00018 (15)
O1	0.0225 (6)	0.0115 (6)	0.0157 (6)	0.0003 (5)	0.0118 (5)	0.0027 (5)
O2	0.0120 (5)	0.0124 (6)	0.0107 (5)	−0.0017 (4)	0.0042 (4)	0.0020 (5)
O3	0.0171 (6)	0.0076 (6)	0.0190 (6)	−0.0005 (5)	0.0094 (5)	−0.0013 (5)
O4	0.0110 (5)	0.0168 (7)	0.0133 (6)	−0.0049 (4)	0.0063 (5)	−0.0040 (5)
O5	0.0163 (6)	0.0141 (6)	0.0103 (6)	−0.0043 (5)	0.0052 (5)	0.0014 (5)
O6	0.0148 (6)	0.0147 (6)	0.0109 (6)	0.0051 (5)	0.0031 (5)	0.0004 (5)
O7	0.0162 (6)	0.0125 (6)	0.0092 (5)	−0.0015 (5)	0.0041 (5)	−0.0030 (5)
O8	0.0139 (6)	0.0134 (6)	0.0210 (6)	−0.0007 (5)	0.0096 (5)	−0.0009 (5)
N1	0.0186 (7)	0.0182 (8)	0.0237 (8)	−0.0020 (6)	0.0129 (7)	−0.0071 (7)
C1	0.0114 (7)	0.0099 (8)	0.0103 (7)	−0.0005 (6)	0.0050 (6)	0.0001 (6)
C2	0.0155 (7)	0.0125 (8)	0.0125 (7)	0.0038 (6)	0.0061 (6)	−0.0007 (7)
C3	0.0181 (8)	0.0156 (9)	0.0180 (9)	0.0017 (6)	0.0111 (7)	−0.0008 (7)

### Geometric parameters (Å, °)

**Table:**

Co1—O2^i^	2.0494 (12)	O1—H1O	0.85 (3)
Co1—O2	2.0494 (12)	O3—H3O	0.76 (3)
Co1—O8^i^	2.1221 (14)	O6—H6O	0.74 (3)
Co1—O8	2.1221 (14)	O8—H81	0.89 (3)
Co1—O5^i^	2.1225 (13)	O8—H82	0.77 (3)
Co1—O5	2.1225 (13)	N1—C3	1.487 (3)
P1—O2	1.5031 (13)	N1—H2N	0.88 (3)
P1—O4	1.5199 (12)	N1—H3N	0.91 (3)
P1—O3	1.5469 (14)	N1—H1N	0.870 (17)
P1—C1	1.8367 (17)	C1—C2	1.542 (2)
P2—O5	1.5115 (13)	C2—C3	1.527 (3)
P2—O7	1.5149 (13)	C2—H2A	0.9900
P2—O6	1.5609 (14)	C2—H2B	0.9900
P2—C1	1.8422 (16)	C3—H3A	0.9900
O1—C1	1.448 (2)	C3—H3B	0.9900
			
O2^i^—Co1—O2	180.0	P2—O5—Co1	130.65 (7)
O2^i^—Co1—O8^i^	92.52 (5)	P2—O6—H6O	111 (2)
O2—Co1—O8^i^	87.48 (5)	Co1—O8—H81	126.4 (17)
O2^i^—Co1—O8	87.48 (5)	Co1—O8—H82	107 (2)
O2—Co1—O8	92.52 (5)	H81—O8—H82	107 (2)
O8^i^—Co1—O8	180.0	C3—N1—H2N	111.7 (18)
O2^i^—Co1—O5^i^	92.85 (5)	C3—N1—H3N	109.5 (17)
O2—Co1—O5^i^	87.15 (5)	H2N—N1—H3N	109 (2)
O8^i^—Co1—O5^i^	90.19 (5)	C3—N1—H1N	107.2 (19)
O8—Co1—O5^i^	89.81 (5)	H2N—N1—H1N	113 (2)
O2^i^—Co1—O5	87.15 (5)	H3N—N1—H1N	106 (2)
O2—Co1—O5	92.85 (5)	O1—C1—C2	108.19 (14)
O8^i^—Co1—O5	89.81 (5)	O1—C1—P1	108.73 (11)
O8—Co1—O5	90.19 (5)	C2—C1—P1	107.85 (11)
O5^i^—Co1—O5	180.00 (7)	O1—C1—P2	109.67 (11)
O2—P1—O4	114.17 (7)	C2—C1—P2	108.63 (12)
O2—P1—O3	110.62 (8)	P1—C1—P2	113.62 (9)
O4—P1—O3	110.93 (8)	C3—C2—C1	113.39 (14)
O2—P1—C1	109.00 (8)	C3—C2—H2A	108.9
O4—P1—C1	105.91 (8)	C1—C2—H2A	108.9
O3—P1—C1	105.73 (8)	C3—C2—H2B	108.9
O5—P2—O7	114.46 (8)	C1—C2—H2B	108.9
O5—P2—O6	111.46 (8)	H2A—C2—H2B	107.7
O7—P2—O6	108.02 (7)	N1—C3—C2	108.60 (15)
O5—P2—C1	107.58 (7)	N1—C3—H3A	110.0
O7—P2—C1	108.58 (8)	C2—C3—H3A	110.0
O6—P2—C1	106.40 (8)	N1—C3—H3B	110.0
C1—O1—H1O	119.0 (17)	C2—C3—H3B	110.0
P1—O2—Co1	129.11 (7)	H3A—C3—H3B	108.4
P1—O3—H3O	115 (2)		
			
O4—P1—O2—Co1	−170.27 (9)	O4—P1—C1—C2	61.76 (13)
O3—P1—O2—Co1	63.77 (11)	O3—P1—C1—C2	179.55 (12)
C1—P1—O2—Co1	−52.07 (12)	O2—P1—C1—P2	58.97 (11)
O8^i^—Co1—O2—P1	−55.98 (10)	O4—P1—C1—P2	−177.76 (9)
O8—Co1—O2—P1	124.02 (10)	O3—P1—C1—P2	−59.97 (11)
O5^i^—Co1—O2—P1	−146.30 (11)	O5—P2—C1—O1	−177.36 (11)
O5—Co1—O2—P1	33.70 (11)	O7—P2—C1—O1	58.25 (13)
O7—P2—O5—Co1	166.92 (9)	O6—P2—C1—O1	−57.80 (13)
O6—P2—O5—Co1	−70.11 (12)	O5—P2—C1—C2	64.58 (13)
C1—P2—O5—Co1	46.17 (13)	O7—P2—C1—C2	−59.81 (13)
O2^i^—Co1—O5—P2	148.83 (11)	O6—P2—C1—C2	−175.86 (11)
O2—Co1—O5—P2	−31.17 (11)	O5—P2—C1—P1	−55.46 (11)
O8^i^—Co1—O5—P2	56.30 (11)	O7—P2—C1—P1	−179.85 (8)
O8—Co1—O5—P2	−123.70 (11)	O6—P2—C1—P1	64.10 (11)
O2—P1—C1—O1	−178.61 (10)	O1—C1—C2—C3	−31.88 (19)
O4—P1—C1—O1	−55.34 (13)	P1—C1—C2—C3	−149.34 (13)
O3—P1—C1—O1	62.45 (12)	P2—C1—C2—C3	87.10 (16)
O2—P1—C1—C2	−61.51 (13)	C1—C2—C3—N1	−173.76 (14)

Symmetry codes: (i) −*x*, −*y*+1, −*z*.

### Hydrogen-bond geometry (Å, °)

**Table:**

*D*—H···*A*	*D*—H	H···*A*	*D*···*A*	*D*—H···*A*
O1—H1O···O5^ii^	0.85 (3)	2.05 (3)	2.845 (2)	155 (2)
O3—H3O···O7^ii^	0.76 (3)	1.72 (3)	2.4627 (19)	167 (3)
O6—H6O···O4^iii^	0.74 (3)	1.78 (3)	2.5124 (18)	175 (3)
O8—H81···O4^iv^	0.89 (3)	1.88 (3)	2.7277 (18)	160 (2)
O8—H82···O1^v^	0.77 (3)	2.25 (3)	2.8953 (19)	142 (3)
N1—H2N···O6^v^	0.88 (3)	2.15 (3)	2.975 (2)	156 (2)
N1—H3N···O2^iv^	0.91 (3)	2.30 (3)	3.096 (2)	146 (2)
N1—H3N···O5^vi^	0.91 (3)	2.32 (3)	3.030 (2)	134 (2)
N1—H1N···O4^vii^	0.87 (2)	2.33 (2)	3.071 (2)	143 (2)

Symmetry codes: (ii) −*x*, *y*+1/2, −*z*−1/2; (iii) *x*−1, −*y*+3/2, *z*−1/2; (iv) −*x*+1, −*y*+1, −*z*; (v) −*x*, *y*−1/2, −*z*−1/2; (vi) *x*+1, *y*, *z*; (vii) −*x*+1, *y*−1/2, −*z*−1/2.
